# Analysis of the stability of 70 housekeeping genes during iPS reprogramming

**DOI:** 10.1038/s41598-020-78863-5

**Published:** 2020-12-10

**Authors:** Yulia Panina, Arno Germond, Tomonobu M. Watanabe

**Affiliations:** 1 RIKEN Center for Biosystems Dynamics Research (BDR), 6-2-3 Furuedai, Suita, Osaka 565-0874 Japan; 2 Hokkaido University Faculty of Pharmaceutical Sciences, Kita Ward, Sapporo, Hokkaido 060-0812 Japan

**Keywords:** Cell biology, Computational biology and bioinformatics, Stem cells

## Abstract

Studies on induced pluripotent stem (iPS) cells highly rely on the investigation of their gene expression which requires normalization by housekeeping genes. Whether the housekeeping genes are stable during the iPS reprogramming, a transition of cell state known to be associated with profound changes, has been overlooked. In this study we analyzed the expression patterns of the most comprehensive list to date of housekeeping genes during iPS reprogramming of a mouse neural stem cell line N31. Our results show that housekeeping genes’ expression fluctuates significantly during the iPS reprogramming. Clustering analysis shows that ribosomal genes’ expression is rising, while the expression of cell-specific genes, such as vimentin (Vim) or elastin (Eln), is decreasing. To ensure the robustness of the obtained data, we performed a correlative analysis of the genes. Overall, all 70 genes analyzed changed the expression more than two-fold during the reprogramming. The scale of this analysis, that takes into account 70 previously known and newly suggested genes, allowed us to choose the most stable of all genes. We highlight the fact of fluctuation of housekeeping genes during iPS reprogramming, and propose that, to ensure robustness of qPCR experiments in iPS cells, housekeeping genes should be used together in combination, and with a prior testing in a specific line used in each study. We suggest that the longest splice variants of Rpl13a, Rplp1 and Rps18 can be used as a starting point for such initial testing as the most stable candidates.

## Introduction

Induced pluripotent stem cells (iPSCs)^1^ are a promising technology that is becoming increasingly important for medical treatment development^2^ and basic research. iPSCs are generated by forced expression of pluripotent genes, such as Oct4, Klf4, Sox2 and Myc, and this process is associated with profound changes in cell metabolism^3^, gene expression, and epigenetics^4^. In the great majority of previous studies, the analysis of gene or protein expression rely on the use of housekeeping genes used as reference for normalization of the data^5^. Typical housekeeping genes relate to the basic functioning of cells, such as cytoskeletal genes (Actb (actin), Tubb5 (tubulin), Vim (vimentin) etc.), ribosomal genes (multiple genes for large and small subunits of the ribosome, such as Rpl7, Rps9 etc.), ATP generation-related genes (such as Gapdh, Pgk1 etc.), various essential enzyme genes (such as fumarate hydratase Fh1, DNA mismatch repair gene Mlh3, Peptidylprolyl Isomerase A (Ppia) etc.), and these genes are generally thought to be stable regardless of the cell state.

The question of stability of housekeeping genes has previously been investigated in mouse embryonic stem cells (mESCs). In 2006, E. Willems and colleagues have identified Actb and Gapdh as the most stable genes in mouse embryos and in differentiating mouse and human ES cells^6^. A subsequent study by S. Mamo in 2007 focused on reference genes in mouse oocytes and embryos, and pointed out the instability of housekeeping genes, while suggesting that Ppia, H2az1 and Hprt were the most stable genes in the embryos^7^. The same team has later published a 2008 study in rabbit oocytes and preimplantation stage embryos. The team identified H2az1, Hprt and Ywhaz as the most stable reference genes, while indicating that Ubc, Tbp and B2m were the least stable and unsuitable for normalization in qPCR experiments in pluripotent stem cells^8^. Another study found that Sdha, Tbp and Ywhaz were the most stable genes during the differentiation of mESCs *in vitro*^9^. A study on human ES cells conducted in 2013, on the other hand, identified B2M and RPL13A as the most stable genes during differentiation^10^. In 2015, another study on differentiating human ES cells expanded the set of analyzed genes because there were concerns about the variability of the housekeeping genes’ expression, and used large-scale datasets to perform global transcriptional analysis using a technique less trusted today, namely microarrays. This study included 9 different datasets and 144 microarrays to identify a set of non-varying genes, while highlighting the fact that commonly used ACTB, GAPDH, HPRT1, PPIA, SDHA and B2M varied substantially during human ESC differentiation. This study put HPRT1 and B2M in the group of highly varied genes^11^. The most notable study conducted in stem cells is the 2007 study by Synnergren and colleagues^12^. This study used microarrays to highlight that conventionally used housekeeping genes such as HPRT1, ACTB and GAPDH fluctuate in differentiating human ESCs. The study has identified a special set of housekeeping genes for use as a reference in pluripotent stem cell experiment but failed to validate the genes by qPCR.

The aforementioned studies have focused on ES cells with the exception of one study, conducted in differentiating iPS cells, where the team investigated the stability of commonly used housekeeping genes^13^. In this study, ACTB, C1orf43, PSMB4, GAPDH and HMBS were identified as the most stable genes out of 16 genes during iPS differentiation. Therefore, currently, there exist no studies on housekeeping genes during the iPS reprogramming process except for a limited study by our team in 2018 that analyzed 13 housekeeping genes^14^. Thus, the stability of common housekeeping genes and the newly suggested housekeeping genes^12^, as well as their performance compared to conventional housekeeping genes, still needs to be investigated in iPS systems, especially during the reprogramming process.

The goal of this work was to check the stability of the majority of all housekeeping genes used in the aforementioned stem cell studies, including the newly suggested genes discovered by Holmgren et.al^11^, so as to provide the most comprehensive list of housekeeping genes as of today. In this work, we have used the most precise method of gene expression quantification^15^, namely RT-qPCR, combined with newly developed high-precision data analysis method, Pairwise Efficiency^16^, to uncover gene expression patterns in 70 housekeeping genes during iPS reprogramming. This work includes all of the most commonly used housekeeping genes as well as recently suggested pluripotency-related genes^12^, and takes into account possible splice variants of the genes. This work uncovers the fluctuations in housekeeping genes during iPS reprogramming and underlines important considerations for the researchers who are using iPS cells in their research to avoid normalization to housekeeping genes without prior check.

## Materials and methods

### Cell culture

The iPS reprogramming was carried out in a reprogrammable cell system previously described in Hikichi et. al., 2012^17^, and applied by Panina and colleagues^14^. The system consists of a mouse neural progenitor cell line named N31 and does not express pluripotency markers nor possesses pluripotent phenotype prior to reprogramming. The “reprogrammability” of this system has been shown in the publications mentioned above. This system enables reprogramming with a doxycycline-inducible cassette with Oct4, Sox2, Klf4 and c-Myc. Addition of doxycycline activates the four factors and initiates reprogramming. The cells were maintained on plastic gelatin-coated dishes (Corning) in RHB neural stem cell media (Takara) supplemented with Ndiff and 1 mg/ml FGF and 1 mg/ml EGF. For reprogramming initiation, the medium was changed to Essential 8 iPS reprogramming medium (home-made) and 1 µg/ml doxycycline was added to the dish. From that point on, the media were changed every day to avoid pH fluctuations. The reprogramming process continued until day 15, and the cell samples were collected on days 0, 5, 10, and 15.

### Gene selection and primer design

Full information about genes, primers and amplicons is available as supplementary data. All primers in this work have been carefully designed to include the longest splice variants of the genes (in contrast to other studies which did not take splicing into account), and have been tailored to work in the same melting temperature using free NCBI tool (https://www.ncbi.nlm.nih.gov/tools/primer-blast/) in order to allow screening of different genes on the same qPCR plate.

### RNA isolation and cDNA synthesis

Total RNA was isolated with RNeasy kit (Invitrogen) according to the manufacturer's instructions, and RNA absorbance ratios (A_260/280_ and A_260/230_) were assessed by Nanodrop 2000 Spectrophotometer (NanoDrop Technologies). 300 ng of RNA from each sample was converted to cDNA using SuperScript First-Strand Synthesis System (Thermo Fisher Scientific). The DNA was assessed by the Nanodrop spectrophotometer and diluted to 100 ng/µl. The reprogramming process (day 0–day 15) was repeated 2 times, thus 2 biological replicates were obtained for each time point, amounting to 18 cell material samples per primer tested. In total, each time point had 2 biological and 4 technical replicas.

### Experiment design and PCR dataset generation

To assess the stability of chosen 70 genes, we gathered total RNA at four time points throughout the process, namely at day 0, day 5, day 10 and day 15. We then performed qPCR of these genes at all four time points using Pairwise Efficiency approach^16^. This approach takes into account the efficiency of qPCR reaction, and is approximately two times more precise compared to conventional Delta Ct method. 4 time points * 70 housekeeping genes resulted in 280 independent DNA-primer combinations. Six replicas were run for each combination, as required by Pairwise Efficiency method, and a total of 1680 amplification curves were obtained. The Baseline Subtracted PCR datasets were generated from each PCR run and processed using Bio-Rad CFX Manager 2.0 (2.0.885.0923). These datasets were imported for analysis into the Pairwise Efficiency software (unpublished).

### Quantitative real-time PCR

qPCR was performed on a CFX96 Connect (BioRad). SYBR Green PCR supermix (BioRad) was used as per the manufacturer's instructions. Each reaction contained 5 ng of cDNA in a final volume of 10 μL, and the primers’ concentration was 300 nM. Thermocycler program was as following: 1) hot start cycle at 95 °C for 3 min, 2) 40 cycles at 95 °C for 10 s, and 3) 60 °C for 30 s. For product specificity confirmation, a melting curve analysis was performed.

### Statistical analyses according to most commonly used algorithms

For selecting the most stably expressed genes we have used the following software freely available, namely, RefFinder^18^ which calculates a comprehensive score from DeltaCt, BestKeeper^19^, NormFinder^20^ and Genorm^21^.

### Statistical analyses and data processing

The data processing was carried out in Microsoft Excel, R, and JMP Version 11 (https://www.jmp.com). Clustering was used to visualize the patterns of variation of gene expression during reprogramming, such as tendencies toward decrease, increase or fluctuation during reprogramming. To cluster the groups of genes that displayed similar tendencies (such as, increasing during reprogramming, or decreasing during reprogramming, or fluctuating), we performed a hierarchical clustering on the gene expression value normalized from 0 to 1 (for each gene). The distances were calculated using the Ward method, which is a well known metric for clustering. Ward’s method joins clusters to maximize the likelihood at each level of the hierarchy. The output of this clustering is presented using two visualizations, a dendrogram in Fig. 2a and a constellation plot in Fig. 2b. In the constellation plot, each housekeeping gene is represented by an endpoint and each cluster join is represented by a new node. The axis scaling, orientation of points, and angles of the lines are arbitrary. However, the lengths of the lines represent the distance between clusters and the lengths are meaningful with respect to each other. The constellation plot is an alternative visualization of the hierarchical clustering dendrogram shown in Fig. 2b and is but another way to look at the statistical distance between clusters. It notably highlights that the first two subgroups (red and green), according to the statistical ward distance calculated between clusters, are classified relatively far apart from the other 5 subgroups, showing that these genes behave significantly differently than the genes of other clusters.

## Results

### The choice of housekeeping genes for the study

In this study, we investigated 70 genes to provide a comprehensive overview of the most of housekeeping genes used in literature (Table 1). The list of 70 genes was assembled using previously published articles for expression patterns in differentiating pluripotent stem cells. The list contains a portion of very commonly used genes as well as genes identified by microarray analysis as stable and suitable for qPCR normalization in differentiating human ES cells^12^. The Supplementary Table S1, available in the supplementary materials, lists gene names, associated symbols and accession numbers, general protein information and splice variants, the functions of the gene products by Gene Ontology, and the mRNA expression in RPKM units obtained in ENCODE transcriptome project for the CNS of mouse embryo (E11.5). The Supplementary Table S2 available in the same excel file lists all the information about primers. All primers were checked for splice variants and were carefully designed to include the longest splice variants to ensure the robustness of the study.

**Table 1:** Summary of 70 housekeeping genes evaluated in this study. Gene symbol, accession number and general protein information are shown.

Symbol	Accession number	General protein information
Aasdh	NM_173765.3	Aminoadipate-semialdehyde dehydrogenase
Actb	NM_007393.5	Actin, beta
Ada	NM_001272052.1	Adenosine deaminase
Alas1	NM_001291835.1	Aminolevulinic acid synthase 1
Alb	NM_009654.4	albumin
Atp5f1	NM_009725.4	ATP synthase, H + transporting, mitochondrial F0 complex, subunit B1
B2m	NM_009735.3	Beta-2 microglobulin
Car6	NM_009802.2	Carbonic anhydrase 6
Cdc14a	NM_001080818.2	CDC14 cell division cycle 14A
Cox4i1	NM_009941.3	Cytochrome c oxidase subunit 4I1
Cpne2	NM_153507.2	Copine II
Crebbp	NM_001025432.1	CREB binding protein
Cript	NM_019936.3	Cysteine-rich PDZ-binding protein
Def8	NM_001253783.1	Differentially expressed in FDCP 8
Dele1	NM_024179.5	DAP3 binding cell death enhancer 1
Dtwd2	NM_026854.3	DTW domain containing 2
Eef1d	NM_029663.2	Eukaryotic translation elongation factor 1 delta (guanine nucleotide exchange protein)
Eln	NM_007925.4	Elastin
Fbxl12	NM_013911.3	F-box and leucine-rich repeat protein 12
Fh1	NM_010209.2	Fumarate hydratase 1
Foxp4	NM_001110824.1	Forkhead box P4
G6pdx	NM_008062.2	Glucose-6-phosphate dehydrogenase X-linked
Gapdh	NM_001289726.1	Glyceraldehyde-3-phosphate dehydrogenase
Got1	NM_010324.2	Glutamic-oxaloacetic transaminase 1, soluble
Gtf2h3	NM_181410.3	General transcription factor IIH, polypeptide 3
Gusb	NM_010368.2	Glucuronidase, beta
H13	NM_001159551.1	Histocompatibility 13
Hddc2	NM_027168.2	HD domain containing 2
Hmbs	NM_001110251.1	Hydroxymethylbilane synthase
Hprt	NM_013556.2	Hypoxanthine guanine phosphoribosyl transferase
Idh3a	NM_029573.2	Isocitrate dehydrogenase 3 (NAD +) alpha
Ldha	NM_001136069.2	Lactate dehydrogenase A
Mdh1	NM_001316675.1	Malate dehydrogenase 1, NAD (soluble)
Mlh3	NM_001304475.1	mutL homolog 3
Mpi	NM_025837.2	Mannose phosphate isomerase
Nubp1	NM_011955.2	Nucleotide binding protein 1
Pdha1	NM_008810.3	Pyruvate dehydrogenase E1 alpha 1
Pfkp	NM_001291071.1	Phosphofructokinase, platelet
Pgam1	NM_023418.2	Phosphoglycerate mutase 1
Pgk1	NM_008828.3	Phosphoglycerate kinase 1
Plekha1	NM_001346515.1	Pleckstrin homology domain containing, family A (phosphoinositide binding specific) member 1
Pole	NM_011132.2	Polymerase (DNA directed), epsilon
Ppia	NM_008907.2	Peptidylprolyl isomerase A
Pten	NM_008960.2	Phosphatase and tensin homolog
Ripk3	NM_019955.2	Receptor-interacting serine-threonine kinase 3
Rnd1	NM_172612.3	Rho family GTPase 1
Rnf7	NM_011279.3	Ring finger protein 7
Rpl13a	NM_009438.5	Ribosomal protein L13A
Rpl15	NM_001359897.1	Ribosomal protein L15
Rpl7	NM_011291.5	Ribosomal protein L7
Rplp1	NM_018853.3	Ribosomal protein, large, P1
Rps11	NM_013725.4	Ribosomal protein S11
Rps18	NM_011296.2	Ribosomal protein S18
Rps3	NM_012052.2	Ribosomal protein S3
Rps9	NM_029767.2	Ribosomal protein S9
Sdha	NM_023281.1	Succinate dehydrogenase complex, subunit A, flavoprotein
Slc4a1ap	NM_001347328.1	Solute carrier family 4 (anion exchanger), member 1, adaptor protein
Slc5a11	NM_146198.2	Solute carrier family 5 (sodium/glucose cotransporter), member 11
Snrpb	NM_009225.2	Small nuclear ribonucleoprotein B
Srp72	NM_025691.1	Signal recognition particle 72
Srsf7	NM_146083.2	Serine/arginine-rich splicing factor 7
Stim1	NM_009287.5	Stromal interaction molecule 1
Tbp	NM_013684.3	TATA box binding protein
Tfrc	NM_011638.4	Transferrin receptor
Tmem41b	NM_153525.5	Transmembrane protein 41B
Tubb5	NM_011655.5	Tubulin, beta 5 class I
Ubc	NM_019639.4	Ubiquitin C
Vim	NM_011701.4	Vimentin
Vsnl1	NM_012038.4	Visinin-like 1
Ywhaz	NM_001253805.1	Tyrosine 3-monooxygenase/tryptophan 5-monooxygenase activation protein, zeta polypeptide

### Reprogramming of mouse neural progenitors into iPS cells

**Figure 1:**
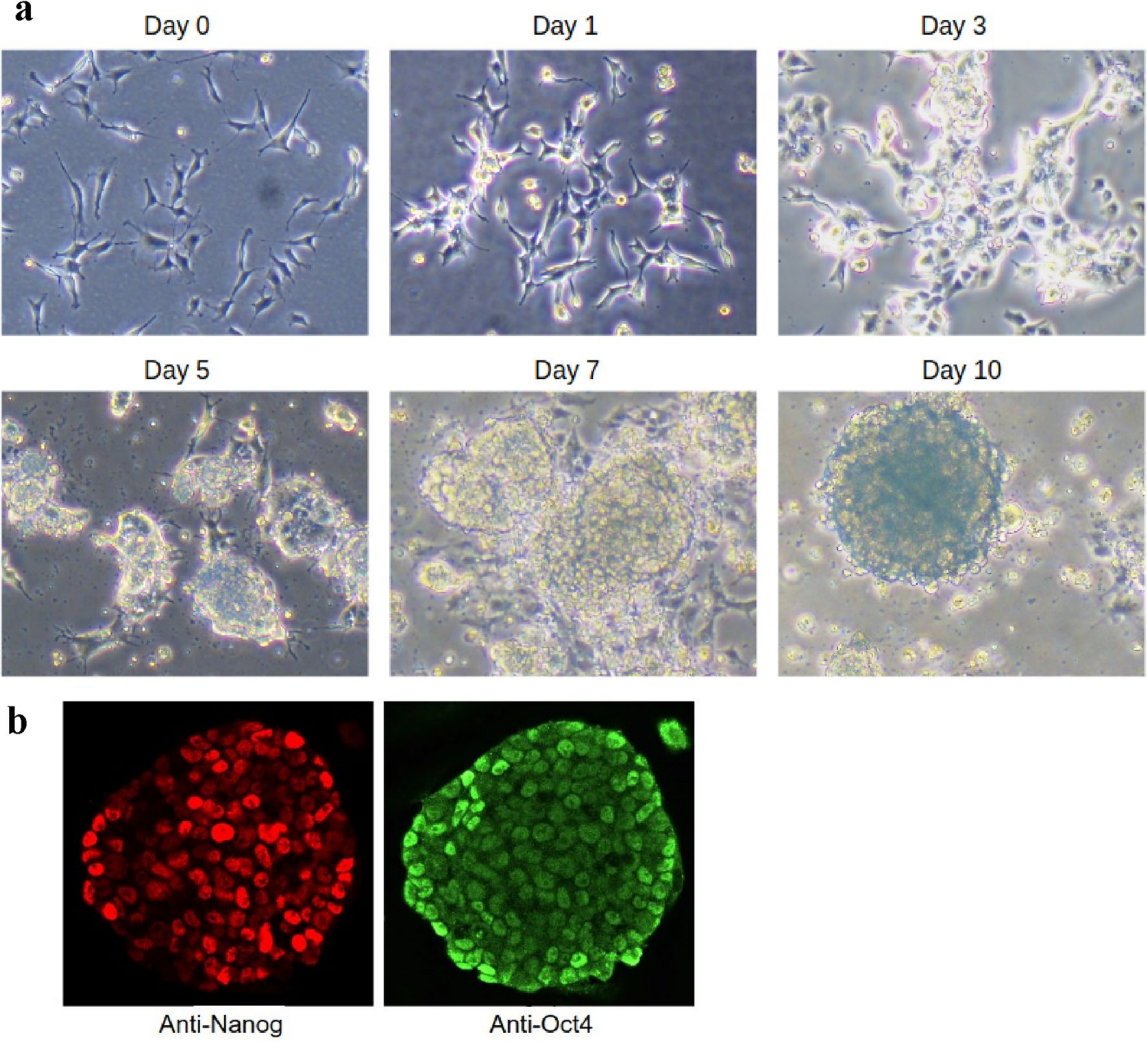
Reprogramming of neural progenitors into the pluripotent iPS cells. **(a)** Colony formation during the iPS reprogramming of neural progenitors. The truly round-shaped colonies with a characteristic “glow” indicative of reprogrammed cells appear between days 7 and 10. (**b)** The results of the immunofluorescent analysis of nascent colonies for pluripotency markers Nanog and Oct4 (Day 10).

To investigate housekeeping genes’ expression changes throughout the reprogramming process, we performed reprogramming of mouse neural progenitors using previously described system^14,17^, and monitored them for standard markers of pluripotency (shape changes and pluripotency markers Nanog and Oct4). The cells began to grow in lumps starting from day 5, and by day 10 they formed characteristic round-shaped colonies with tight edges. Immunostaining for pluripotency markers Nanog and Oct 4 showed that the colonies fully expressed these genes at day 10 (Fig. 1) (the markers were absent in the parental neural progenitors, data not shown).

### Housekeeping genes fluctuate during iPS reprogramming

**Figure 2:**
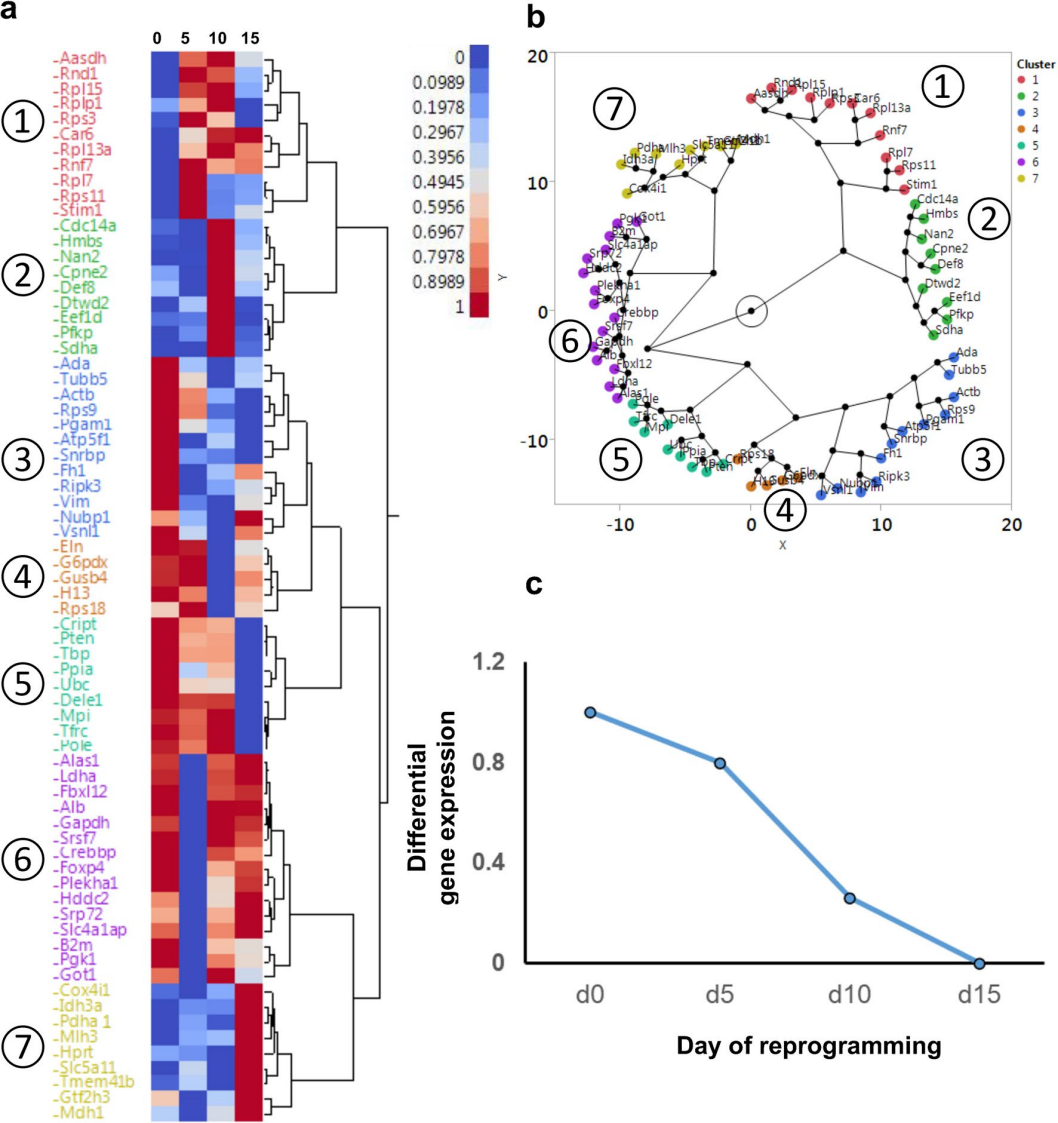
Expression change of 70 housekeeping genes throughout the iPS reprogramming process. **(a)** Clustering analysis of the dynamics of gene expression levels during reprogramming. Gene expression levels relative to Day 0 were considered and scaled from 0 to 1 for each gene. The color scale attributes red to a value of 1, indicating the highest gene expression during reprogramming. Four time points during the reprogramming are indicated over the heatmap (Days zero, five, ten and fifteen), and the gene names are shown on the left. JMP Version 11 software was used to create the figure (https://www.jmp.com). **(b)** Alternative visualization of the clustering analysis of **(a)** using a constellation plot. Genes are grouped by their similarity of expression dynamics. Lengths of the lines represent the statistical distance between clusters. **(c)** Relative dynamic of Actb gene expression by comparison to Day 0 during reprogramming.

The stability of the 70 candidate genes was monitored at four time points throughout the reprogramming process, and gene expression values were calculated by Pairwise Efficiency approach. To reveal patterns in the expression fluctuation of the genes, the expression was scaled from 0 to 1 across the four time points. Data were then submitted to a hierarchical clustering to visualize the group of genes that behaved similarly during reprogramming (Fig. 2). The analysis revealed three major types of patterns, and a total of 7 subgroups (clusters). This analysis (Fig. 2a,b) clearly placed together most of the ribosomal genes such as Rpl15, Rplp1, Rpl13a, Rpl7, Rps3 and Rps11 (cluster 1), and they displayed a rising pattern, especially on Day 5 and Day 10 of the reprogramming. Cluster 2 contained some of the biosynthetic genes (such as Hmbs), cell cycle genes (such as Cdc14a), and other genes with unclear function (such as Def8), showing a fluctuating pattern. Clusters 3 and 4 were separated by a small statistical distance (represented by the length of the branches on Fig. 2b) and contained cytoskeleton-related genes, such as Actin (Actb), Tubulin (Tubb5), Elastin (Eln) and Vimentin (Vim), as well as two small ribosomal subunits Rps9 and Rsp 18. The mRNA expression of these genes tended to fall throughout the process of iPS reprogramming. Clusters 6 and 7 contained genes related to growth (specifically, ATP production) and cell cycle, such as Gapdh, Ldha, Mdh1, Pgk1, Got1, Foxp4, Pdha, Idh3a, Mlh3 and Cox4i1. Most of the genes in this group are involved in the steps of glucose breakdown and ATP generation (e.g. Gapdh is a glyceraldehyde-3-phosphate dehydrogenase which turns Glyceraldehyde 3-phosphate into 1,3-bisphosphoglyceric acid, and Pgk1 is the next step in the chain, converting 1,3-bisphosphoglyceric acid into 3-Phosphoglyceric acid). The genes in this group were found to fluctuate (rise and fall) during the reprogramming.

### Assessing the degree of change in individual genes

**Figure 3:**
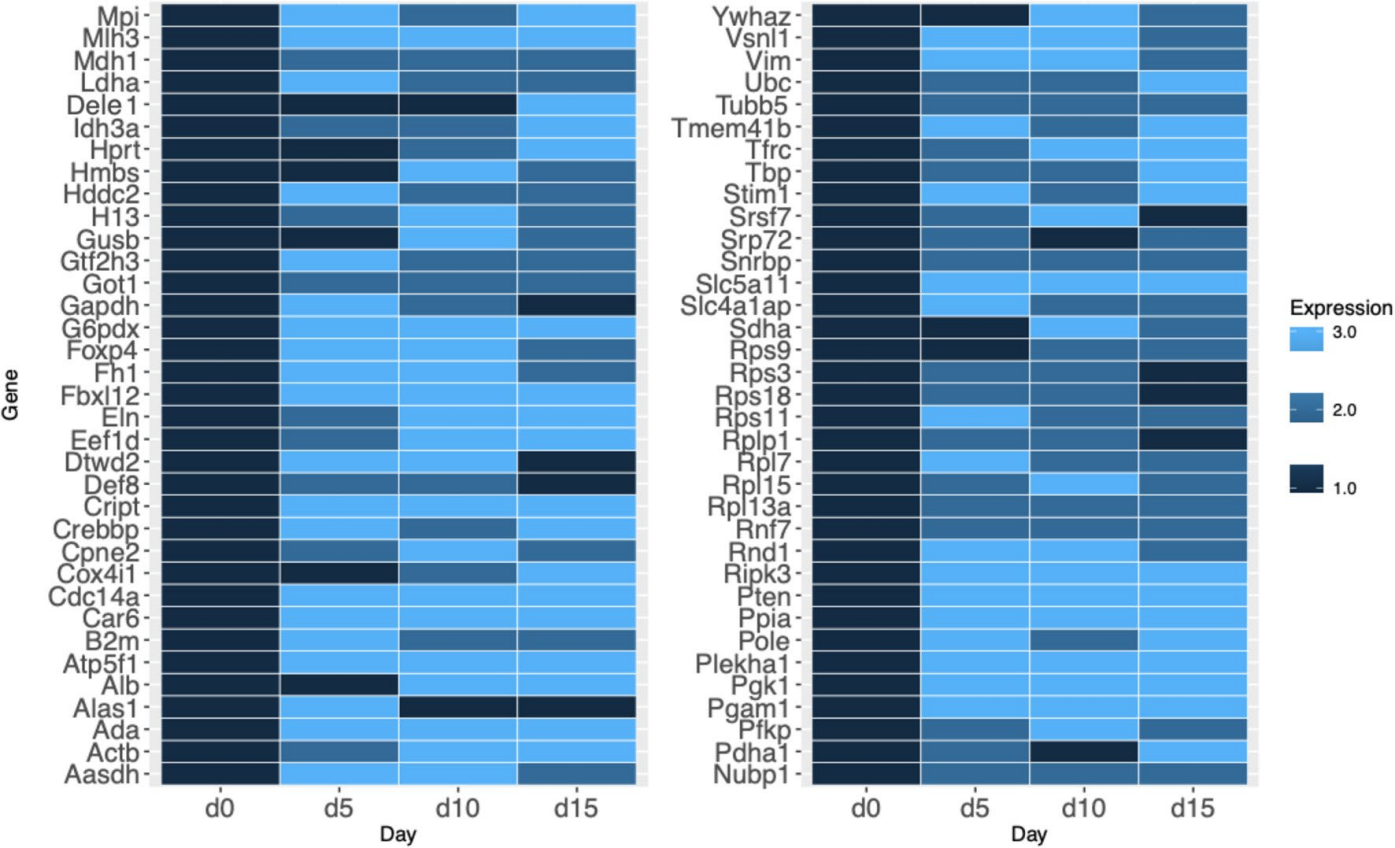
A heatmap representing fold changes in the housekeeping genes’ expressions throughout the iPS reprogramming process. The genes are grouped by fold-expression change into one of three groups, represented by dark blue, medium blue and light blue. Four time points during the reprogramming are indicated under the heatmaps, and the gene names are written on the left. The dark blue color represents no expression change, and Day 0 is taken as the “time point zero” before initiation of reprogramming. The medium blue color represents the expression change of more than statistically significant 1.5-fold, but less than threefold. Light blue represents gene expression change greater than threefold, compared to time point zero (Day 0).

To assess the degree of change of the individual genes and reveal which genes are the most stable overall during the iPS reprogramming, we calculated the relative gene expression by comparison to Day 0. Then, we associated 3 colors to the gene levels to reflect the following: (1) The expression level equal to that of Day 0 (i.e., no change compared to non-reprogrammed cells), dark blue, (2) the expression level at least 1.5 times greater but less than threefold (transcripts showing a |FC|≥ 1.5 and a False Discovery Rate (FDR) < 0.05), medium blue, and (3) gene expression change of more than threefold, light blue. We found that all genes showed significant changes in the expression levels, and there was not a single gene that would show constant (dark blue only) expression during the whole process of reprogramming (Fig. 3). Moreover, 80% of all genes displayed changes in gene expression levels that were more than three-times fold at least on one of the days of reprogramming (light blue tiles). Only 20% of all genes stayed within the three-times fold change in expression levels (no light blue tiles), among them Tubulin (Tubb5), ribosomal subunits Rsp3, Rpl13a and Rsp18 and less known housekeeping genes Snrpb and Srp72. Importantly, this visualization showed that the most popular reference genes, Actb, Gapdh, Hprt, Ppia and B2m belonged to the group which fluctuated more than threefold. Since for the purposes of normalization it is necessary that the reference gene does not change its expression, we compiled a list of genes that stayed relatively constant, that is, within the category 1 and category 2 (dark blue and medium blue tiles) at all four time points during the iPS reprogramming process (Table 2).

**Table 2:** Selected genes that do not change their expression more than three-fold during the iPS reprogramming.

Mouse gene symbol	Human gene symbol	Gene name
Tubb5	TUBB	Tubulin beta class I
Rps3	RPS3	Ribosomal protein S3
Rsp18	RPS18	Ribosomal protein S18
Rplp1	RPLP1	Ribosomal protein lateral stalk subunit P1
Rpl13a	RPL13A	Ribosomal protein L13a
Mdh1	MDH1	Malate dehydrogenase 1
Got1	GOT1	Glutamic-oxaloacetic transaminase 1
Srp72	SRP72	Signal recognition particle 72
Snrpb	SNRPB	Small nuclear ribonucleoprotein polypeptides B and B1
Rnf7	RNF7	Ring finger protein 7
Nubp1	NUBP1	Nucleotide binding protein 1
Def8	DEF8	Differentially expressed in FDCP 8

### The most stably expressed genes during iPS reprogramming

**Figure 4:**
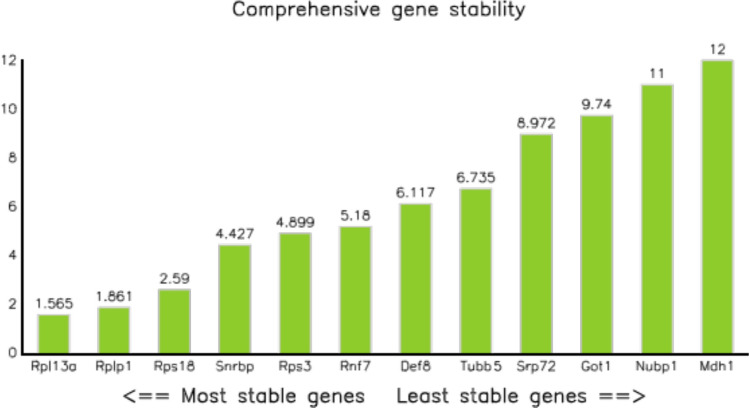
The most stable genes out of 70 selected for this study. The most stable genes are shown on the left, and the least stable genes are shown on the right. The small ribosomal subunit 13a is ranked as the most stable by all algorithms, with the large ribosomal subunit Rplp1 following it. Overall, the ribosomal subunits Rpl13a, Rplp1 and Rps18 were selected as the most stable genes throughout the iPS reprogramming.

Finally, we subjected the selected twelve genes (Table 2) to the comprehensive ranking analysis that takes into account most of existing stability analyses software available, namely, RefFinder which calculates a comprehensive score from DeltaCt, BestKeeper, NormFinder and Genorm (see “Materials and methods”), and determined the most stable gene overall using the software (Fig. 4). Rpl13a was ranked the most stable overall, following by Rplp1 and Rps18.

## Discussion

iPS cells are increasingly used for basic research, medical applications and drug testing. Since the research using these cells progresses rapidly, and more and more works include RNA sequencing data or qPCR data that rely on housekeeping genes for normalization, it is very important to know whether such normalization is suitable in iPS cells and specifically during the reprogramming process. In this work, we have analyzed the stability of 70 housekeeping genes throughout the reprogramming of mouse neural progenitors. Genes were selected from all known literature, to our best knowledge, making this study the first comprehensive comparison of stability in housekeeping genes. Importantly, we found that all housekeeping genes changed their expression more than two-fold during the iPS reprogramming. Moreover, 80% of the genes changed more than three-fold (Fig. 3). While these results were obtained in only one cell line, namely, mouse neural progenitors, it illustrates several important points: (1) housekeeping genes should not be assumed to be stable until actually tested, (2) iPS reprogramming affects even the cell maintenance-related genes, (3) therefore, more studies in other cell lines may uncover differences between species and cell lines.

It was of interest to us to see how the pattern of gene expression changed across different genes, for which we employed clustering analysis. Clustering approach revealed patterns of gene expression dynamics (Fig. 2). Ribosomal genes, which appeared in the same cluster, had a tendency to increase their expression throughout the reprogramming. This result is in agreement with the concept of iPS reprogramming, where the change in pluripotency state from less pluripotent to more pluripotent is associated with the increase in cell cycle progression and speed^22–24^. The increase in cell cycle speed would logically be associated with the increased need for ribosomal RNA synthesis. Interestingly, the analyses revealed that all ribosomal genes were grouped together, suggesting their temporal dynamics is similar during reprogramming. To validate this hypothesis, further investigations (for example, the measurements of nucleoli size or modern RNA staining techniques) would be needed. It is also worth noting that, while most ribosomal genes were found to increase during the iPS reprogramming process, some ribosomal genes displayed a fluctuating pattern (e.g. Rps18 and Rps9). Both of these genes belong to the small ribosomal subunit. Hypothetically, these two genes might play a distinctive role in regulating the production of ribosomes and by that, regulate the cell growth and division state. Indeed, recent research has also pointed at such a possibility, simulating the behavior of ribosomal genes and identifying them as a major speed-regulating hub for cell cycle progression^25^.

It is important to note that, in the current work, we have used a different strategy for primer design compared to our previous publication (Panina et al., 2018). In the present study, we conducted an analysis of splice variants of each gene (Sup. Table S1), and found that several genes possessed different splice variants. In the present work, we designed all primers so as to include the longest splice variant (Sup. Table S2). For example, the Rps18 gene in the present work includes the Exon 1 at the beginning of the gene sequence. This could be one explanation why the Rps18 gene in the current work was among the most stable ones, while in the previous publication (Panina et.al., 2018), where we used primer pairs that corresponded to the central exon of Rps18 gene (Sup. Figure), Rps18 was among the least stable ones. We thus caution the reader to use the same primers when attempting to reproduce results from other publications.

Our clustering analysis also grouped several ATP-generating genes into two categories: the genes whose expression decreases following a similar pattern (decreasing genes), and the genes whose expression fluctuates throughout the reprogramming. For example, such genes as Pgk1 and Pgam1 were grouped together as decreasing. Interestingly, Pgk1 and Pgam1 represent consequent steps in glycolysis. Pgk1 is a phosphoglycerate kinase, a glycolytic enzyme involved in step 2 of the pathway and catalyzes the conversion of 1,3-diphosphoglycerate to 3-phosphoglycerate, and Pgam1 catalyzes the reaction of 3-phosphoglycerate to 2-phosphoglycerate. On the other hand, Pfkp, Idh3a and Pdha1 were clustered next to each other on the other, “increasing” part of the clustering spectrum. Pfkp is the platelet-specific isoform of phosphofructokinase and plays a key role in glycolysis regulation, Idh3a is catalytic subunit of isocitrate dehydrogenase and catalyze the allosterically regulated rate-limiting step of the tricarboxylic acid (TCA) cycle. Pdha is a subunit of pyruvate dehydrogenase E1, a mitochondrial multienzyme complex that provides the primary link between glycolysis and the TCA cycle. TCA cycle is a process happening in mitochondria and is related to oxidative phosphorylation (OXPHOS). Since iPS reprogramming is well-known to involve profound changes in the balance between glycolysis and OXPHOS^3^, it would be interesting to research glycolytic genes further to shed more light on the control of these events.

It should also be noted that the current study focuses on the mouse neural stem cells and cannot claim that the results would be the same in other cell lines. Different cell lines may differ in the expression of the possible variants of housekeeping genes. As a case in point, MiniSox9 is a shorter splice variant of the Sox9 gene, and acts as a silencer of the full‐size (the longest) splice variant of Sox9, which can lead to drastic differences in the actual Sox9 expression depending on cell line and primers^26^. We thus caution the reader to investigate the actual performance of their primers of choice in their particular cell line while keeping in mind which splice variants would be amplified. This study should serve as a general guideline for the possible genes that can be tested.

In the end, we have evaluated all selected genes for their relative stability during the iPS reprogramming. Our results showed that the longest splice variants of Rpl13a, Rplp1 and Rps18 were the most stable during the process, which makes them the best candidates for normalization during RNA experiments. Overall, our analysis has shown that all currently known housekeeping genes fluctuate during the iPS reprogramming, and that the best strategy would be to combine several genes. We have suggested candidates for that.

## Supplementary Information

Supplementary Figure 1.

Supplementary Tables.

## Data Availability

All data concerning genes and primers are available in the Supplementary Material.
