# Supplementary material for: Analysis of the stability of 70 housekeeping genes during iPS reprogramming
Source: Sci Rep. 2020 Dec 10;10:21711. doi: 10.1038/s41598-020-78863-5 (PMC7728746; doi:10.1038/s41598-020-78863-5)
Supplement: Supplementary file 1 — Supplementary Figure 1. [file 41598_2020_78863_MOESM1_ESM.pdf]

Analysis of the stability of 70 housekeeping genes during iPS reprogramming

Yulia Panina[1,2,3*], Arno Germond[1], Tomonobu M. Watanabe[1]

[1]RIKEN Center for Biosystems Dynamics Research (BDR), 6-2-3 Furuedai, Suita, Osaka 565-0874, Japan
[2]Kyoto University Center for iPS Cell Research and Application (CiRA), 53 Shogoin Kawaharacho, Sakyo Ward, Kyoto, 606-8507, Japan
[3]Hokkaido University Faculty of Pharmaceutical Sciences, Kita Ward, Sapporo, Hokkaido 060-0812, Japan

*Corresponding author: Yulia Panina, Hokkaido University Faculty of Pharmaceutical Sciences, Kita Ward, Sapporo, Hokkaido 060-0812, Japan
Email: yulia.panina@pharm.hokudai.ac.jp

SUPPLEMENTARY FIGURE

a

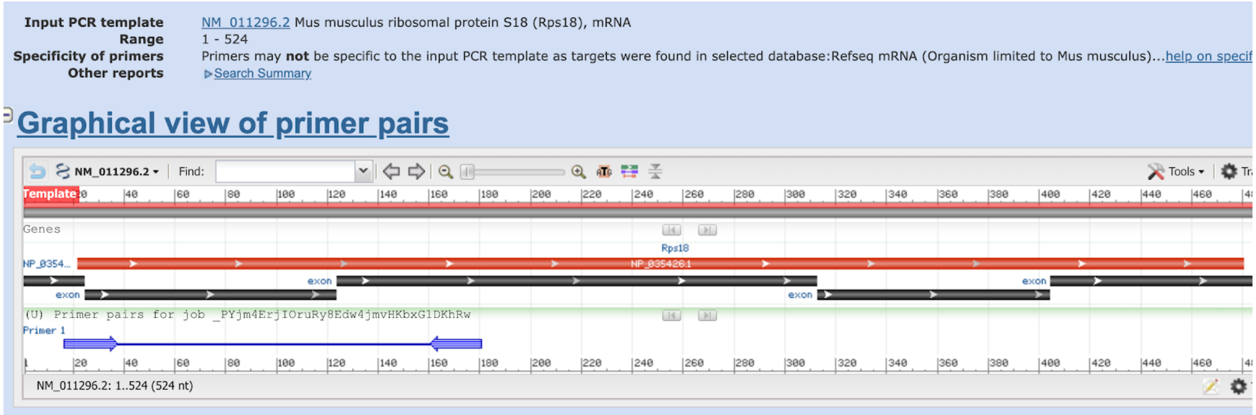

b

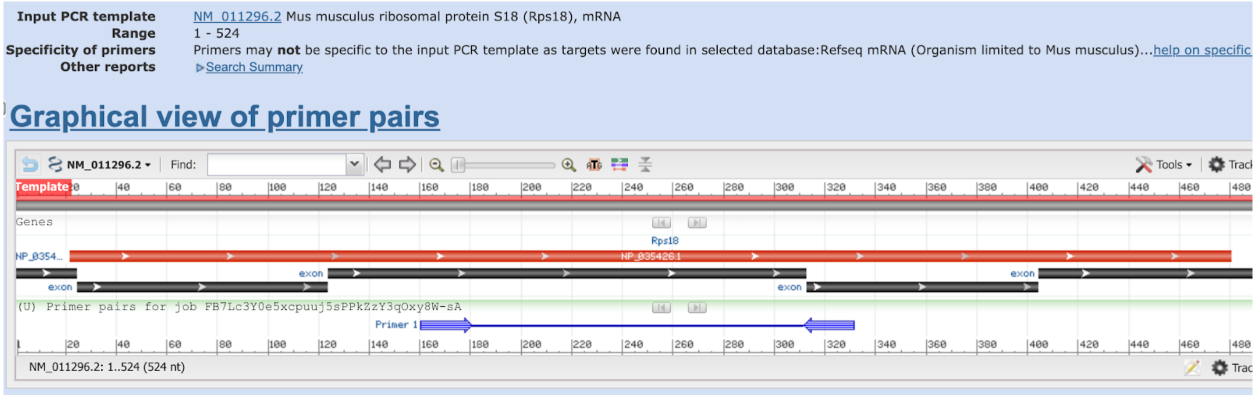

Supplementary Figure. Differences in primer design and the resulting PCR products for the gene Rps18. A. Primers in the current study are designed to pick up the first three exons and correspond to the longest splice variant of Rps18. B. Primers in the 2018 study were designed to pick up only the central exon, and may not have reflected the behavior of the longest splice variant.
